# Novel variants of *ABCA4* in Han Chinese families with Stargardt disease

**DOI:** 10.1186/s12881-020-01152-5

**Published:** 2020-10-31

**Authors:** Fang-Yuan Hu, Feng-Juan Gao, Jian-kang Li, Ping Xu, Dan-Dan Wang, Sheng-Hai Zhang, Ji-Hong Wu

**Affiliations:** 1 Eye Institute and Department of Ophthalmology, Eye & ENT Hospital, Fudan University, Shanghai, China; 2 NHC Key Laboratory of Myopia (Fudan University); Key Laboratory of Myopia, Chinese Academy of Medical Sciences, Shanghai, China; 3 Shanghai Key Laboratory of Visual Impairment and Restoration, Shanghai, China; 4 BGI-Shenzhen, Shenzhen, China; 5 Department of Computer Science, City University of Hong Kong, Kowloon, Hong Kong

**Keywords:** *ABCA4* gene, STGD1, Han Chinese patients, Panel-based NGS, Novel mutant variants

## Abstract

**Background:**

Stargardt disease (STGD1) is a common recessive hereditary macular dystrophy in early adulthood or childhood, with an estimated prevalence of 1:8000 to 1:10,000. *ABCA4* is the causative gene for STGD1. The current study aims at identifying the novel disease-related *ABCA4* variants in Han Chinese families with STGD1 using next-generation sequencing (NGS).

**Methods:**

In the present study, 12 unrelated Han Chinese families (19 males and 17 females) with STGD1 were tested by panel-based NGS. In order to capture the coding exons and the untranslated regions (UTRs) plus 30 bp of intronic flanking sequences of 792 genes, which were closely associated with usual ophthalmic genetic disease, we designed a customized panel, namely, Target_Eye_792_V2 chip. STGD1 patients were clinically diagnosed by experienced ophthalmologists. All the detected variants were filtered and analyzed through the public databases and in silico programs to assess potential pathogenicity.

**Results:**

Twenty-one *ABCA4* mutant variants were detected in 12 unrelated Han Chinese families with STGD1, containing 14 missense, three splicing, two frameshift, one small deletion, and one nonsense variants. Base on the American College of Medical Genetics (ACMG) guidelines, 8 likely pathogenic and 13 pathogenic variants were determined. The functional consequences of these mutant variants were predicted through in silico programs. Of the 21 mutant variants in *ABCA4*, two novel coding variants c.3017G > A and c.5167 T > C and one novel null variant c.3051-1G > A were detected in three unrelated probands.

**Conclusions:**

By panel-based NGS, 21 *ABCA4* variants were confirmed in 12 unrelated Han Chinese families. Among them, 3 novel mutant variants were found, which further expanded the *ABCA4* mutation spectrum in STGD1 patients.

**Supplementary Information:**

The online version contains supplementary material available at 10.1186/s12881-020-01152-5.

## Background

*ABCA4,* mapping to chromosome 1p22.1, encodes the ATP-binding cassette transporter 4, which is a transmembrane protein highly expressed in the cone and rod photoreceptors [1, 2]. This transmembrane protein acts as a flippase and mainly regulates the transmembrane transport of N-retinylidene-phosphatidylethanolamine across the disc membranes in the outer segments of cone and rod. The dysfunction of the ABCA4 protein results in the massive accumulation of A2E, a by-product of the visual cycle, in the retinal pigment epithelium (RPE) as lipofuscin deposits, which induces RPE apoptosis and secondary photoreceptor degeneration [3–6]. The mutant variants in *ABCA4* are mainly responsible for autosomal recessive retinal dystrophies, including Stargardt disease (STGD1; OMIM 248200) [1, 7], retinitis pigmentosa (RP19; OMIM 601718) [8, 9], cone-rod dystrophy (CRD3; OMIM 604116) [10, 11], and early-onset severe retinal dystrophy (OMIM 248200) [12, 13]. According to the Human Gene Mutation Database (HGMD), more than 1200 disease-associated *ABCA4* variants are already found in retinopathy, including missense, nonsense, splicing, frameshift, small insertion or deletion, and gross insertion or deletion variants. Among them, approximately 80% of variants in *ABCA4* are associated with STGD1.

STGD1 is an inherited macular atrophic disease in childhood or early adulthood, following an autosomal recessive inheritance pattern [12, 14]. It is typically characterized by central visual impairment and macular atrophy [14–17]. The underlying gene for STGD1 is *ABCA4* [1]. In our previous study, we have completed *ABCA4* gene screening among a large Chinese population with STGD1 to extend the *ABCA4* mutation spectrum and identified three prevalent *ABCA4* variants, namely, c.101_106delCTTTAT, c.2894A > G, and c.6563 T > C [18]. Although many *ABCA4* variants have been reported, we found that novel *ABCA4* mutant variants were still found in STGD1 patients.

In the present study, we analyzed the mutation spectrum of *ABCA4* in 12 Han Chinese families with STGD1 by next-generation sequencing (NGS). Our analysis revealed 21 *ABCA4* mutant variants and the functional impacts of these variants were evaluated by in silico programs. Among them, 3 novel mutant variants were determined, namely, two missense and one splicing variants.

## Methods

### Subjects

From June 2019 to October 2019, 36 subjects from 12 unrelated Han Chinese families with STGD1 were recruited at Fudan University Eye Ear Nose and Throat Hospital. Ophthalmologic examination was performed on the subjects, containing spectral domain optical coherence tomography (SD-OCT), electroretinography (ERG), fundus autofluorescence (FAF), slit-lamp biomicroscopy, fundus photograph, and best corrected visual acuity (BCVA). In addition, the family and medical histories were recorded. STGD1 patients were clinically diagnosed by experienced ophthalmologists.

### Library preparation and panel-based NGS

We cooperated with BGI-Shenzhen to design a specially customized capture panel, named as Target_Eye_792_V2 chip, which could capture the exons and the untranslated regions (UTRs) plus 30 bp of intronic flanking sequences of 792 genes closely associated with usual ophthalmic genetic disease (Supplementary Table S1). In the current study, all subjects underwent corresponding genetic testing by panel-based NGS. The genomic DNA of all subjects was extracted from peripheral blood by QIAGEN FlexiGene DNA Kit (Qiagen, Venlo, the Netherlands) according to the manufacturer’s protocols. Then, the genomic DNA was sonicated to shear into fragments, including the promoter and flanking intronic regions and the coding exons. The enriched libraries were sequenced by BGISEQ-500 [19].

### Genetic analyses and variant identification

Sequencing reads that passed quality control were aligned to the reference sequences (UCSC hg 38) through the Burrows-Wheeler Aligner (BWA) program [20]. The reference sequence number for *ABCA4* is NM_000350. We analyzed the minor allele frequencies (MAFs) of all identified mutant variants in the four databases, namely, ExAC (http://exac.broadinstitute.org), dbSNP (http://www.ncbi.nlm.nih.gov/projects/SNP/), ESP6500 (http://evs.gs.washington.edu/EVS/), and 1000 Genomes Project (http://browser.1000genomes.org/). The variants with MAFs> 0.1% were filtered out to eliminate benign variants. After filtering, the mutant variants were further screened according to variant report, the potential deleterious effects, and genotype-phenotype correlation in the three major databases, including OMIM (http://www.omim.org/), HGMD (http://www.hgmd.cf.ac.uk/ac/index.php), and ClinVar (https://www.ncbi.nlm.nih.gov/clinvar/) [21, 22]. Variants were determined as likely pathogenic or pathogenic based on the American College of Medical Genetics (ACMG) guidelines. Candidate variants were validated by Sanger sequencing.

## Results

### Clinical findings

**Fig. 1:**
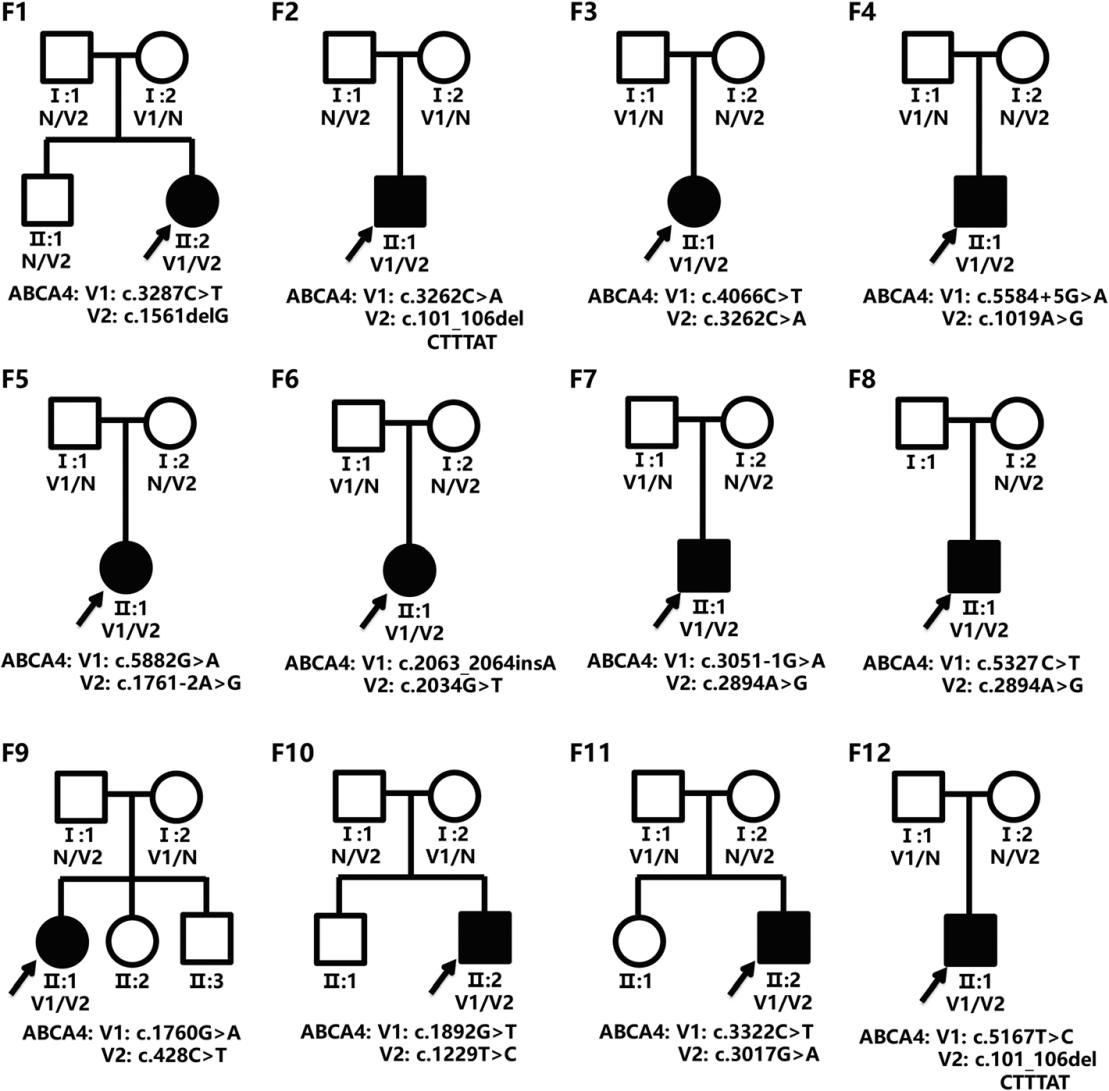
Pedigrees of 12 Han Chinese families and identified mutants. Circles and squares indicate females and males, respectively. The darkened shapes indicate the affected members and the empty shapes indicate the subjects with normal phenotype. “N” represents a wild type allele and “V” represents a mutant allele

**Fig. 2:**
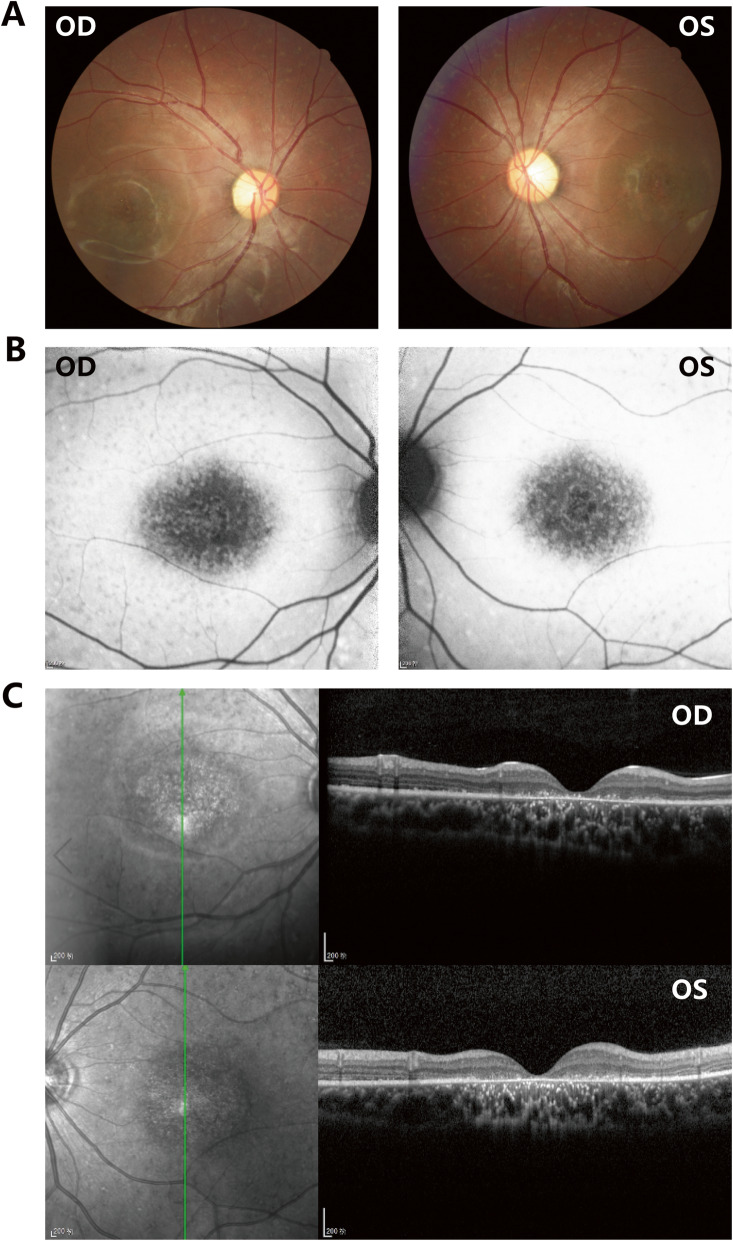
The representative images of ophthalmologic examination from proband F1:II:2. **a** Fundus photographs exhibiting macular atrophy and yellow-white spots in the posterior pole. **b** FAF exhibiting macular hypo-autofluorescence corresponding with atrophy and multiple hyper-fluorescent dots corresponding with subretinal flecks. **c** OCT exhibiting the significant thinning of retinal outer layers in macular area

Twelve unrelated Chinese families (19 males and 17 females) with STGD1 underwent genetic testing by panel-based NGS in this study. The basic clinical phenotypic characteristics of 12 probands were shown in Table 1. The vast majority of probands showed early onset age, mainly in early childhood or adolescence. Among them, the longest disease duration was more than 20 years. The visual acuity of probands was significantly decreased in both eyes, and some patients complained of an increased difficulty in adapting to darkness and abnormal color vision. In addition, all parents showed the normal phenotypes, and the pedigrees of 12 families were obtained (Fig. 1). Fundus examination in probands revealed the atrophy of the bilateral RPE around the macula. Representative photographs of patient F1:II:2 were shown in Fig. 2. Fundus photographs showed some pigment mottling and yellow-white flecks in bilateral macula and a beaten-bronze macular appearance (Fig. 2a). Fundus autofluorescence (FAF) displayed the fluorescence blocking due to macular pigment mottling and the multiple hyper-fluorescent dots corresponding with subretinal flecks detected in fundus photographs (Fig. 2b). Macular OCT revealed macular atrophy, reduced thickness of the retinal outer layers, and the alteration of choroidal reflectivity (Fig. 2c).

**Table 1:** The *ABCA4* disease-associated variants identified in this study

ProbandID	Age (years)	Sex	Age of onset (years)	BCVA (OD/OS)	Exon/ Intron	Nucleotide change	Amino acid change	Variant type	Zygosity	References
F1:II:2	19	Female	10	0.15/0.15	Exon22	c.3287C > T	p.(Ser1096Leu)	Missense	Het	Stone et al. (2017)
					Exon12	c.1561delG	p.(Val521Serfs*47)	Frameshift	Het	Wang et al. (2018)
F2:II:1	31	Male	10	0.05/0.02	Exon22	c.3262C > A	p.(Pro1088Thr)	Missense	Het	Hu et al. (2019) [18]
					Exon2	c.101_106delCTTTAT	p.(Ser34_Leu35del)	Inframe Deletion	Het	Huang et al. (2018)
F3:II:1	8	Female	2	0.05/0.1	Exon27	c.4066C > T	p.(Gln1356*)	Nonsense	Het	Hu et al. (2019) [18]
					Exon22	c.3262C > A	p.(Pro1088Thr)	Missense	Het	Hu et al. (2019) [18]
F4:II:1	22	Male	15	0.06/0.06	Intron39	c.5584 + 5G > A	p.[Thr1821Aspfs*6,Thr1821Valfs*13]	Splicing	Het	Sangermano et al. (2018)
					Exon8	c.1019A > G	p.(Tyr340Cys)	Missense	Het	Nassisi et al. (2018)
F5:II:1	39	Female	34	0.15/0.05	Exon42	c.5882G > A	p.(Gly1961Glu)	Missense	Het	Wiszniewski et al. (2005)
					Intron13	c.1761-2A > G	p.?	Splicing	Het	Jiang et al. (2016) [23]
F6:II:1	28	Female	23	0.3/0.2	Exon14	c.2034G > T	p.(Lys678Asn)	Missense	Het	Zaneveld et al. (2015)
					Exon14	c.2063_2064insA	p.(Asn688Lysfs*78)	Frameshift	Het	Jiang et al. (2016) [23]
F7:II:1	28	Male	3	0.07/0.05	Exon19	c.2894A > G	p.(Asn965Ser)	Missense	Het	Zernant et al. (2011)
					Intron21	c.3051-1G > A	p.?	Splicing	Het	This study
F8:II:1	22	Male	7	0.01/0.1	Exon19	c.2894A > G	p.(Asn965Ser)	Missense	Het	Zernant et al. (2011)
					Exon38	c.5327C > T	p.(Pro1776Leu)	Missense	Het	Mandal et al. (2005)
F9:II:1	10	Female	8	0.1/0.5	Exon12	c.1760G > A	p.(Arg587Lys)	Missense	Het	Fujinami et al. (2015) [14]
					Exon4	c.428C > T	p.(Pro143Leu)	Missense	Het	Consugar et al. (2015) [21]
F10:II:2	20	Male	10	0.2/0.15	Exon13	c.1892G > T	p.(Gly631Val)	Missense	Het	Hu et al. (2019) [18]
					Exon9	c.1229 T > C	p.(Ile410Thr)	Missense	Het	Jiang et al. (2016) [23]
F11:II:2	13	Male	11	0.3/0.6	Exon22	c.3322C > T	p.(Arg1108Cys)	Missense	Het	Sodi et al. (2006)
					Exon20	c.3017G > A	p.(Gly1006Asp)	Missense	Het	This study
F12:II:1	16	Male	14	0.05/0.1	Exon36	c.5167 T > C	p.(Tyr1723His)	Missense	Het	This study
					Exon2	c.101_106delCTTTAT	p.(Ser34_Leu35del)	Inframe Deletion	Het	Huang et al. (2018)

*Abbreviations*: *BCVA* best corrected visual acuity, *OD* right eye, *OS* left eye. [*] represents termination codon

### Detected variants and genetic analyses

Twenty-one disease-related *ABCA4* variants were identified in 12 Han Chinese families, containing 18 known and 3 novel mutant variants (Fig. 1, Table 1). As presented in Supplementary Table S2, the detailed genetic analyses and the functional evaluation of 21 distinct variants were summarized. Two mutant variants were identified in all probands and all of them were identified as compound heterozygotes. Except for the parent F8: I: 1 who was not detected, all parents of the proband were confirmed to possess one *ABCA4* variant and clinically diagnosed as normal phenotype. In addition, the elder brother of the proband F1:II:2 also possessed one heterozygous variant, while the other siblings with the normal phenotype in families 9, 10, and 11 did not receive genetic testing.

The detected *ABCA4* variants contained 14 missense, three splicing, two frameshift, one small deletion, and one nonsense variants. The pathogenicity of the variants was determined based on the ACMG guidelines, including 8 likely pathogenic and 13 pathogenic variants. In our previous studies, we have confirmed three prevalent *ABCA4* variants in STGD1 patients from China [18]. Among them, two frequent variants, c.101_106delCTTTAT and c.2894A > G, were detected in four families in the present study. In families 2 and 12, variants c.3262C > A and c.5167 T > C were found in trans with c.101_106delCTTTAT, respectively. Variants c.3051-1G > A and c.5327C > T were found in a compound heterozygous state with c.2894A > G in families 7 and 8, respectively. Furthermore, c.3262C > A, c.4066C > T, and c.1892G > T, which were first identified by us in STGD1 patients [18], were also found in three families, namely, families 2, 3, and 10.

**Fig. 3:**
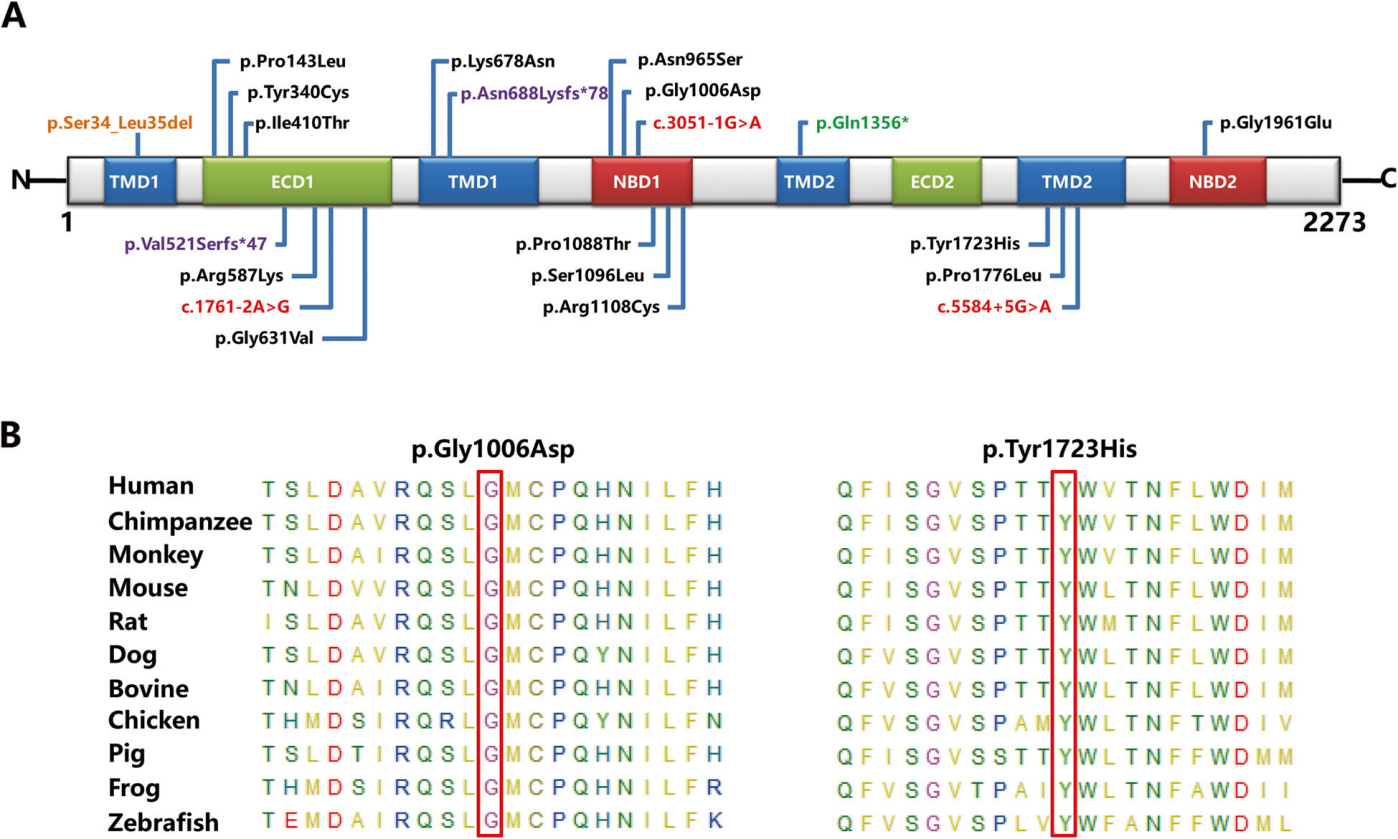
Distribution of the detected mutants in the ABCA4 functional domains and conservation analyses. **a** Location of 21 mutants in six functional domains. Black: missense mutant. Red: splicing mutant. Purple: frameshift mutant. Green: nonsense mutant. Yellow: small deletion mutant. **b** Multiple orthologous sequence alignment displaying the conserved amino acid residues (Glycine 1006 and Tyrosine 1723)

Of the 21 mutant variants, 18 detected variants were distributed in 12 exons of the *ABCA4* gene. And in introns 12, 21, and 39 of *ABCA4*, three splicing variants were identified. Based on the analyses of the Human Splicing Finder (HSF), c.1761-2A > G in intron 12 and c.3051-1G > A in intron 21 mainly affected splice acceptor sites. Variant c.5584 + 5G > A in intron 39 could affect splice donor sites (Supplementary Table S2). Moreover, our analyses revealed that the identified variants were mainly located in exocytoplasmic domains 1 (ECD1) and nucleotide-binding domain 1 (NBD1) (Fig. 3a). The NBD1 domain was regarded as one of the high incidence regions of variants in STGD1 patients from Chinese population in our previous study [18].

### Novel variants in the *ABCA4* gene

Three novel *ABCA4* mutant variants were identified in three unrelated families, namely, one splicing variant and two missense variants (Table 1). Two novel missense variants, c.3017G > A p.Gly1006Asp and c.5167 T > C p.Tyr1723His, were detected in families 11 and 12, respectively. In family 11, variant c.3017G > A, located in the NBD1 domain, was found in trans with c.3322C > T in proband F11:II:2. In proband F12:II:1, variant c.5167 T > C, located in transmembrane domain 2 (TMD2), was detected in compound heterozygosity with c.101_106delCTTTAT (Table 1, Fig. 3a). As presented in Fig. 3b, glycine residue at position 1006 and tyrosine residue at position 1723 were highly conserved in the ABCA4 protein across different species, suggesting that these two variants, p.Gly1006Asp and p.Tyr1723His, might result in a deleterious effect. In accordance with the ACMG guidelines, two novel heterozygous variants were categorized as likely pathogenic variants. Moreover, one novel spicing variant c.3051-1G > A was found in proband F7:II:1 and detected in trans with c.2894A > G. On the basis of the HSF analyses, c.3051-1G > A was predicted to affect the normal splicing. Variant c.3051-1G > A was identified as likely pathogenic in accordance with the ACMG guidelines.

## Discussion

In the current study, 21 *ABCA4* mutant variants were found in 12 unrelated Han Chinese families with STGD1 by genetic testing. Among them, three novel variants were identified.

Except F5: II: 1, the onset age of all probands with STGD1 was relatively early. The mean onset age in 12 families was 12.25 years (range 2–34 years), which was younger than that of the previously reported patients with STGD1. The average age of onset in these reported cohorts is mostly around 20 years [18, 24, 25]. However, some studies have also shown that the onset age of STGD1 patients is earlier, with the average age of onset between 10 and 14 years [23, 26, 27].

In our previous research, c.101_106delCTTTAT p.Ser34_Leu35del, c.6563 T > C p.Phe2188Ser, and c.2894A > G p.Asn965Ser have been confirmed to be three prevalent *ABCA4* variants in STGD1 patients mainly from eastern China [18]. Interestingly, two frequent variants c.101_106delCTTTAT and c.2894A > G were also detected in 4 of 12 unrelated families in the present study. Based on the fact that all of the 12 families also come from eastern China, such results further illustrate the high frequency of the two mutant variants in this region. Our analyses have revealed that the allele frequencies of these three prevalent variants are about 20% [18], which are lower than those of some high frequency variants in STGD1 patients from Europe. For instance, the frequencies of p.Gly1961Glu and p.[Leu541Pro;Ala1038Val] may exceed 30% in European populations [25, 28]. Meanwhile, in another study of *ABCA4* gene screening for STGD1 patients in Chinese population, the frequencies of the prevalent variants (namely, c.101_106delCTTTAT, c.2424C > G, c.2894A > G, and c.6563 T > C) are actually only 14% [23]. According to the above findings, we speculated that the prevalence of STGD1 in China might be lower than that in European populations. So far, there is no exact statistical data on the STGD1 prevalence in Chinese populations. It is necessary to conduct the epidemiological investigation on a larger number of patients in the future, so as to better understand the disease characteristics of STGD1 in Chinese patients.

In addition, the common mutant variant c.5882G > A p.Gly1961Glu identified in eastern Africa and Europe [28, 29] was also detected in family 5, and c.1761-2A > G was detected in trans with c.5882G > A in proband F5:II:1. It has been reported that STGD1 cases harboring the p.Gly1961Glu allele tend to have a mild disease phenotype, and patients with variant p.Gly1961Glu in either the homozygous or heterozygous states show delayed onset of symptoms and later age of onset. Moreover, p.Gly1961Glu is closely related to the bull’s eye maculopathy phenotype of STGD1 [30–32]. Consistent with previous researches, the proband F5:II:1 carrying p.Gly1961Glu presented the highest age of onset (34 years) among all patients and the FAF imaging showed typical bull’s eye maculopathy.

Furthermore, variants c.3262C > A, c.4066C > T, and c.1892G > T that were first confirmed by our group [18] were also detected in three unrelated families in this study. In proband F10:II:2, c.1892G > T was found in compound heterozygosity with c.1229 T > C and both variants were located in the ECD1 domain. Variant c.3262C > A was detected in two families. In particular, c.3262C > A and c.4066C > T were detected in proband F3:II:1 who was identified as compound heterozygotes. These results further indicated the potential deleteriousness caused by the three *ABCA4* mutants.

Of the 21 disease-associated *ABCA4* mutants detected in the current study, 18 known variants were detected in all probands, including 12 missense, two splicing, two frameshift, one small deletion, and one nonsense mutants. Among them, 16 known mutants were distributed in 12 exons of the *ABCA4* gene. Analysis revealed three dissimilar mutants in exon 22; two dissimilar mutants each in exons 12 and 14; and one mutant each in exons 2, 4, 8, 9, 13, 19, 27, 38, and 42. In addition, two known splicing mutants were identified in two introns of *ABCA4* (introns 12 and 39). As presented in Supplementary Table S2, the function prediction of coding variants was performed by four software prediction programs, including MutationTaster, SIFT, FATHMM, and LRT. Through the analyses with four online tools, the functional deleteriousness of 12 known missense mutants was verified by at least three of the four prediction programs. Three severe/null variants, namely, one nonsense mutant c.4066C > T and two frameshift mutants c.1561delG and c.2063_2064insA, could introduce a premature truncating codon in the protein during translation. The three severe/null variants and one inframe deletion variant c.101_106delCTTTAT were predicted to have deleterious effects through the MutationTaster analyses. Moreover, two known splicing variants, c.5584 + 5G > A and c.1761-2A > G, mainly affect the splice donor site and the splice acceptor site, respectively. The detected known variants of *ABCA4* were distributed in all protein functional domains except for the exocytoplasmic domain 1 (ECD1). On basis of the online tools analyses and ABCA4 protein structure, p.Lys678Asn and p.Pro1776Leu located in the TMD1 and TMD2 domains might disrupt the transmembrane alpha-helices and give rise to the dysfunction of ABCA4 protein transport. Variants p.Pro143Leu, p.Tyr340Cys, p.Ile410Thr, p.Arg587Lys, and p.Gly631Val in the ECD1 domain might have an impact on the topologically associated domains outside the cell and lead to the loss of protein function. Variants p.Gly1961Glu, p.Arg1108Cys, p.Ser1096Leu, p.Pro1088Thr, and p.Asn965Ser in the NBD1 and NBD2 domains could affect the ATP hydrolysis function of protein through damaging the topologically associated domains in cells. The functional impacts of p.Asn965Ser have been demonstrated in the corresponding in vivo and in vitro studies [33, 34]. The pathogenicity of these variants was determined based on the ACMG guidelines, containing 5 likely pathogenic and 13 pathogenic mutants.

Three novel *ABCA4* variants were found in this study, including one splicing and two missense variants. All three mutants showed the dramatically low allelic frequency in dbSNP, 1000 Genomes Project, ExAC, and ESP6500 databases and were not observed in 200 normal controls. Two novel missense mutants, c.3017G > A p.Gly1006Asp and c.5167 T > C p.Tyr1723His, co-segregated with the clinical phenotype of STGD1 in families 11 and 12. In silico analyses of missense variants revealed that c.3017G > A and c.5167 T > C could cause the functional damage in all of four software prediction programs (MutationTaster, LRT, SIFT, and FATHMM). Meanwhile, all of these two variants were detected in trans with the known pathogenic variants (c.3322C > T and c.101_106delCTTTAT), further indicating their possible pathogenicity. More significantly, c.3017G > A was located in a high incidence region of variants, namely, the NBD1 domain [18, 23]. Moreover, codons 1006 and 1723 of *ABCA4* were strictly conserved amino acids among dissimilar species, suggesting that these amino acids were essential for the normal protein function. According to the ABCA4 protein structure and the online tools analyses, p.Gly1006Asp in the NBD1 domain might destroy the topologically associated domains in cells, thus interfering with the ATPase activity of the protein. And p.Tyr1723His in the TMD2 domain might bring about the ABCA4 transport dysfunction through breaking the extracellular topological domains. A novel null mutant, c.3051-1G > A in intron 21, was identified and co-segregated with the clinical phenotype of disease in family 7. As shown in Fig. 1, c.3051-1G > A was found in trans with c.2894A > G, which has been confirmed to be a high frequency variant in Chinese populations [18, 23]. According to HSF analyses, variant c.3051-1G > A affects the splice acceptor site. The above-mentioned analysis results confirmed the potential pathogenicity of these three novel mutants. In accordance with the ACMG guidelines, three novel mutants were categorized as likely pathogenic variants.

In the process of screening patients with the clinical phenotype of STGD1, we also detected the variants of other pathogenic genes instead of ABCA4 related to macular degeneration in another three Chinese families, namely, GUCY2D (c.2513G > A), PDE6C (c.967 T > C and c.1579C > T), and POC1B (c.1153G > A and c.458C > T). All of them are the causative genes for cone or cone-rod dystrophy [35–37]. The phenotypes of macular degeneration caused by these three genes are very similar to STGD1. The results also fully demonstrate the importance of genetic testing in clinical differential diagnosis.

## Conclusions

In conclusion, 21 disease-related *ABCA4* variants were identified in 12 unrelated Han Chinese families with STGD1, and the functional influences of the detected mutants were assessed by the software prediction programs. Furthermore, two novel missense and one novel splicing variants were found in three unrelated probands, extending the mutation spectrum of *ABCA4* in patients from Chinese population. The detailed genetic characterization of STGD1 patients will contribute to the clinical differential diagnosis of macular degeneration and provide reliable information for genetic counselling.

## Supplementary Information

**Additional file 1: Table S1.** Gene list of capture panel.

**Additional file 2: Table S2.** Twenty-one *ABCA4* variants identified in this study and their in silico functional assessment.

## Data Availability

Our data generated and/or analysed during the current study is available in the ABCA4 LOVD (www.lovd.nl/ABCA4), which is an almost complete variant and case registry for ABCA4. Here is the link: https://databases.lovd.nl/shared/individuals?search_owned_by_=%3D%22Fangyuan%20Hu%22

## Abbreviations

AMD: Age-related macular degeneration\
ACMG: American College of Medical Genetics
BCVA: Best corrected visual acuity
BWA: Burrows-Wheeler Aligner
CRD: Cone-rod dystrophy
ERG: Electroretinography
ECD: Exocytoplasmic domain
FAF: Fundus autofluorescence
HGMD: Human Gene Mutation Database
HSF: Human Splicing Finder
MAFs: Minor allele frequencies
NGS: Next-generation sequencing
NBD: Nucleotide-binding domain
RPE: Retinal pigment epithelium
RP: Retinitis pigmentosa
STGD1: Stargardt disease
SD-OCT: Spectral domain optical coherence tomography
TMD: Transmembrane domain
UTRs: Untranslated regions
